# Dosimetric impact of stopping power for human bone porosity with dual-energy computed tomography in scanned carbon-ion therapy treatment planning

**DOI:** 10.1038/s41598-024-68312-y

**Published:** 2024-07-29

**Authors:** Masashi Yagi, Yushi Wakisaka, Jun Takeno, Shintaro Kanada, Toshiro Tsubouchi, Noriaki Hamatani, Hiroyasu Maruo, Masaaki Takashina, Takayoshi Ishii, Tatsuaki Kanai, Shinichi Shimizu, Kazuhiko Ogawa

**Affiliations:** 1 Department of Carbon Ion Radiotherapy, Osaka University Graduate School of Medicine, Osaka, Japan; 2 Department of Radiation Technology, Osaka Heavy Ion Therapy Center, Osaka, Japan; 3 Department of Radiotherapy, Medical Co. Hakuhokai, Osaka Proton Therapy Clinic, Osaka, Japan; 4 Department of Medical Physics and Engineering, Osaka University Graduate School of Medicine, Osaka, Japan; 5 Department of Medical Physics, Osaka Heavy Ion Therapy Center, Osaka, Japan; 6 Department of Radiation Oncology, Osaka University Graduate School of Medicine, Osaka, Japan

**Keywords:** Radiotherapy, Applied physics

## Abstract

Few reports have documented how the accuracy of stopping power ratio (SPR) prediction for porous bone tissue affects the dose distribution of scanned carbon-ion beam therapy. The estimated SPR based on single-energy computed tomography (SECT) and dual-energy CT (DECT) were compared for the femur of a Rando phantom which simulates the porosity of human bone, NEOBONE which is the hydroxyapatite synthetic bone substitute, and soft tissue samples. Dose differences between SECT and DECT were evaluated for a scanned carbon-ion therapy treatment plan for the Rando phantom. The difference in the water equivalent length was measured to extract the SPR of the examined samples. The differences for SPR estimated from the DECT-SPR conversion were small with − 1.8% and − 3.3% for the Rando phantom femur and NEOBONE, respectively, whereas the differences for SECT-SPR were between 7.6 and 70.7%, illustrating a 1.5-mm shift of the range and a dose difference of 13.3% at maximum point in the evaluation of the dose distribution. This study demonstrated that the DECT-SPR conversion method better estimated the SPR of the porosity of bone tissues than SECT-SPR followed by the accurate range of the carbon-ion beams on carbon-ion dose calculations.

## Introduction

A growing number of researchers are interested in using heavy-charged particles as cancer treatment modalities, particularly carbon ions. These ions have beneficial properties like a good depth-dose profile known as the “Bragg peak,” where low levels of energy are deposited in proximal region to the target, and the majority of energy is released in the target, little lateral scattering, and increased biological potency near the Bragg peak. These qualities are enhanced for carbon-ion therapy by three-dimensional (3D) pencil-beam scanning^1^. There is a growing number of heavy ion centers worldwide to take advantage of the unique physical and radiobiological properties in the treatment of a variety of malignancies^2^. Recently, it has been recommended to use carbon-ion beams at an ultrahigh dose rate to boost the treatment effectiveness^3,4^.

The dose distribution is frequently calculated in carbon-ion therapy treatment planning using single-energy kV X-ray computed tomography (CT). It is possible to calculate the water-equivalent length for the pencil-beam model using effective-depth calculations with longitudinal heterogeneity corrections. By using the CT number-to-stopping power ratio (SPR) curve, the effective depth is calculated by integrating the stopping power with respect to the water along the beam's path linearly. This differs from photon radiotherapy (i.e. CT number-to-relative-electron density)^5^. Therefore, to determine the range of a carbon-ion beam and generate accurate dose distributions, particularly for a nonhomogeneous geometry, an accurately calibrated CT number-to-SPR curve is necessary.

The CT number-to-SPR curve is often created using the stoichiometric calibration method^6^. However, a single CT number cannot differentiate between a change in density or the chemical composition of an imaged material^7^ because photons and particles interact differently with matter^8,9^. A change in CT number can happen when the X-ray spectrum is varied because of the difference between patient size and calibration phantom (i.e., beam-hardening effect)^10^. Errors in estimation of SPR can occur when the chemical composition of a patient does not match that of the calibration tissue set because of differences in age, sex, diet, or health state^8^. Therefore, the bijection between the CT numbers and the SPR values can never be exact. Dual-energy CT (DECT) can coherently outperform the single-energy CT (SECT) for SPR accuracy. SPRs obtained with DECT are more robust in situations where the chemical composition of the patient tissue differs from that of the calibration tissue set^8^.

The use of nontissue equivalent material for this purpose may induce substantial bias and misleading conclusions^11^. The uncertainty in prediction of SECT-SPR and DECT-SPR depends on empirical knowledge of the radiological properties of human tissue, and the validation and comparison of SPR prediction methods in real tissue is therefore indispensable. SPR prediction has been validated with SECT^12–15^ and DECT^16–19^ for animal tissues. However, these experimental methods have limited applicability for heterogeneous tissue, and in particular, for bone, which has cortical and trabecular compartments that comprise a porous structure consisting of low- (e.g., air), middle- (e.g., marrow), or high-density tissue (e.g., calcium). The skeleton is a unique organ system in the body because it is composed of a calcified tissue (namely, bone) that consists of approximately 60% inorganic component (hydroxyapatite), 10% water, and 30% organic component (bone matrix proteins)^20^. Accurate SPR prediction for bone is crucial for carbon-ion therapy because bone constitutes one of the largest organs in mammals and is distributed throughout the whole body with functional heterogeneity^21,22^, and carbon-ion beams must often pass through bone tissues during treatment^23,24^. SECT- and DECT-based treatment plans have been mostly evaluated for proton therapy^25–28^. Currently, few reports have documented the effect of the accuracy of SPR prediction for porous bone tissue (i.e., large density difference in bone tissue) on the dose distribution of scanned carbon-ion beam therapy.

Consequently, this study sought to validate SPR conversion methods derived from SECT and DECT for estimating SPR values in porous bone tissues and the effect of the estimated SPR accuracy on the dose distribution of scanned carbon-ion beam therapy.

## Methods

### Conversion from CT data to SPR

SPR for DECT was calculated via DEEDZ-SPR conversion^29^. The SPR of each voxel was calculated from the relative electron density (RED, ρ_e_) and mean excitation energy using the Bethe–Bloch formula:

1 $$\begin{array}{c}SPR = {\rho }_{e}\left[1-\frac{ln\frac{I}{{I}_{w}}}{ln\left\{\frac{2{m}_{e}{c}^{2}{\beta }^{2}}{{I}_{w}\left(1-{\beta }^{2}\right)}-{\beta }^{2}\right\}}\right]\end{array}$$

where m_e_ is electron mass, c is the speed of light, and β is the velocity relative to the speed of light. I and I_w_ are the mean excitation energies of the material and water, respectively; an I_w_ = 75.3 eV^30^ was used. The particle velocity relative to the speed of light in a vacuum was 0.481 (131.0 MeV/u)^31^. ρ_e_ in the equation can be obtained using a linear relation with respect to energy-subtracted CT numbers:

2 $$\begin{array}{c}{\rho }_{e} = a\frac{\left(1 + \alpha \right){HU}_{H}-{\alpha HU}_{L}}{1000} + b\end{array}$$

where HU_k_ is the CT number in Hounsfield units for high- or low-kV (k = H or L, respectively) scans. The parameters a, b, and α were obtained by least-squares fitting of ρ_e_ of MODEL 062M (CIRS, Inc., Norfolk, VA, USA) inserts.

The mean excitation energy I was calibrated against the effective atomic number (EAN, Z_eff_) for soft-tissue and bone-tissue inserts with a separation point at EAN = 8.8:

3 $$\begin{array}{c}ln\frac{I}{{I}_{w}} = {c}_{1}^{soft/bone}\left[{\left(\frac{{Z}_{eff}}{{Z}_{eff},w}\right)}^{m}-1\right]-{c}_{0}^{soft/bone}\end{array}$$

Two sets of ${c}_{1}^{soft/bone}$ and ${c}_{0}^{soft/bone}$ were the fitting parameters for soft-tissue and bone-tissue inserts, respectively, and used with the same values previously determined^29^. I was determined by using the Bragg additivity rule.

The following formula was used to estimate EAN by fitting the ratio of EAN of MODEL 062M inserts to that of water as a function of the reduced CT number ${\mu }_{L} = \frac{{HU}_{L}}{1000} + 1$, and RED:

4 $$\begin{array}{c}{\left(\frac{{Z}_{eff}}{{Z}_{eff},w}\right)}^{m}-1 = {\gamma }_{L}\left(\frac{{\mu }_{L}}{{\rho }_{e}}-1\right)\end{array}$$

where γ_L_ was obtained by least-squares fitting. EAN was evaluated by Mayneord’s equation with an exponent of m = 3.3.

Stoichiometric calibration^6^ was used to determine the CT number-to-SPR curve for SECT. Using MODEL 062M, a CT number-to-SPR table was created that was used by the treatment planning system (TPS) to estimate the SPR ratios for SECT-based carbon-ion treatment planning. Insert compositions in the phantom, which are used for CT number calibration, were provided by the vendor. MODEL 062M consists of 2 nested disks that enable us to simulate head and body regions. The phantom configuration used for the CT number calibration was changed depending on the size of the test materials (i.e. scan field of view (SFOV) size). The tissues used to calculate the SPR values were adopted from representative tissues^32^, according to a previous publication 110 of the International Commission on Radiological Protection. The CT number-to-SPR curves were shown in Supplementary Figure S1.

All image processing was conducted with in-house software created using Python (version 3.6.10).

### SPR measurements

**Figure 1:**
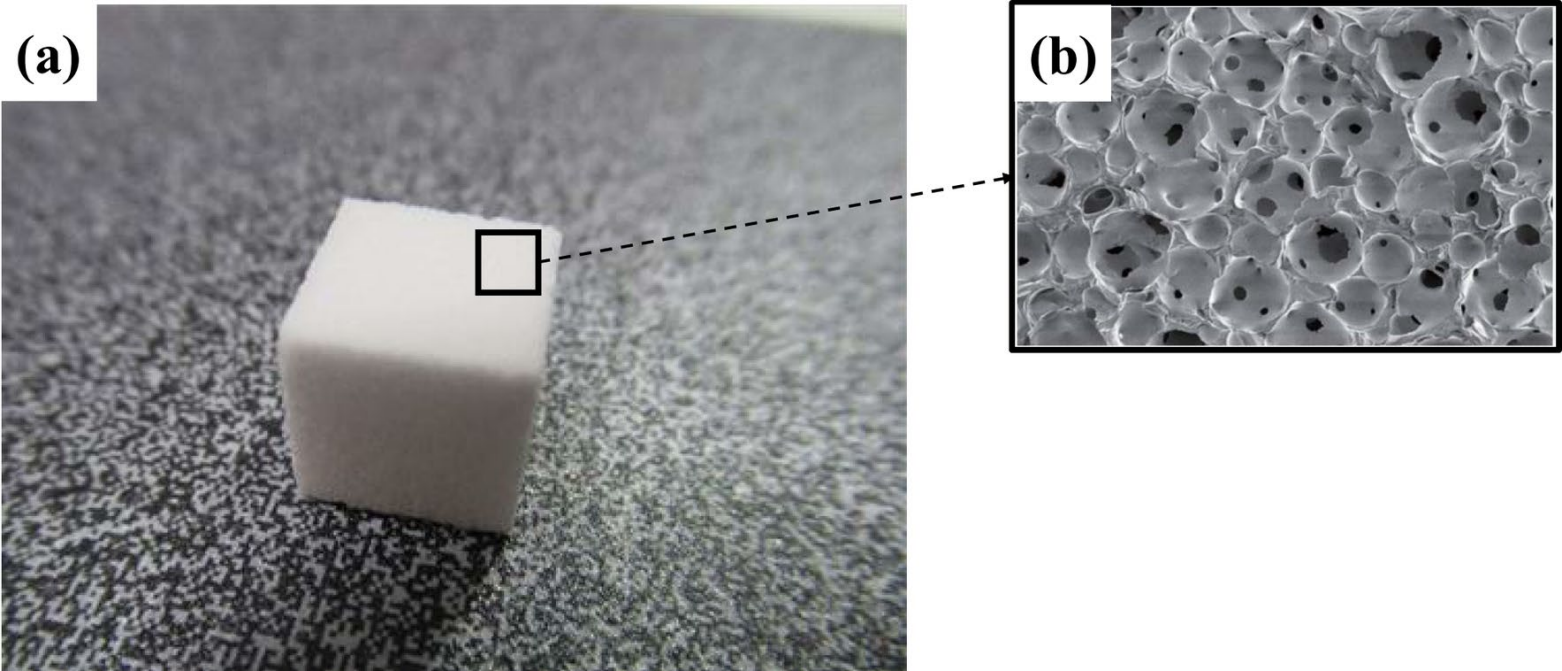
(**a**) A view of NEOBONE used for SPR measurements. (**b**) An electronic microscope view of NEOBONE surface showing the three-dimensional and abundant inter-connected pore structure (supplied by Aimedic MMT).

The accuracy of the SPR conversion methods derived from SECT and DECT was investigated using lean meat and fatty meat of fresh beef which are separately packed in a different petri dish (Falcon, no. 353004; Becton Dickinson, Franklin Lakes, United States; diameter 60 mm and height 15 mm), the femur of the Rando phantom (Radiological Support Devices, Inc., CA, USA), and NEOBONE® (BN01-004, Aimedic MMT, Tokyo, Japan) as test materials. The test samples were one each. Careful attention was paid not to create air pockets in soft tissue samples (i.e. lean meat and fatty meat) to avoid uncertainties caused by any such pockets, and this was checked in the CT images. The lean and fatty meats were put inside stacked acryl plates of which size is comparable dimensions in-plane to MODEL 062M used for the calibrations. Early versions of the Rando phantom contain a human skeleton in a tough plastic that is based on a synthetic isocyanate rubber^33^. The Rando Phantom is transected-horizontally into slices. One of the slices with embedded the femur was used for the measurement. The incident point of the beam was marked on the slice with a marker pen and localized with a room laser. NEOBONE® consists of a calcium hydroxyapatite ceramic and has a 3D and abundant inter-connected pore structure that is designed with 75% porosity in volume, an 150-μm average pore diameter, and an 40-μm average inter-pore-connection diameter^34^ (Fig. 1).

The SPR value of each material was measured as previously described^35^. The SPR of each material was measured using the following equation.

5 $$\begin{array}{c}SPR=\frac{{R}_{water}-{R}_{insert}}{{L}_{insert}}\end{array}$$

where, *R*_*water*_, *R*_*insert*_ and *L*_*insert*_ are the range in water, range in material, and physical length of the material, respectively. The range was determined at the depth of the 90% dose level against the maximum dose (R90). The range of a 302.1 MeV/u carbon-ion beam was measured using a StingRay (IBA Dosimetry Gmbh, Schwarzenbruck, Germany) and an accordion-type water phantom (AVWP03, Accelerator Engineering Corporation, Chiba, Japan). The expanded uncertainty (k = 1) of the measured SPR was estimated as 0.5%. This is due to the uncertainty of the measured dose for R90.

The cylindrical region of interest (diameter 4 mm, length 9 mm in anterior–posterior direction) was applied for the extraction of the SPR from the SECT- and DECT-SPR maps. The mean value of the region of interest was used as the estimated SPR value. The uncertainties of the SPR extracted from SECT and DECT were estimated in our imaging system. The details of the estimation method can be found in 36 and 37 for SECT, and 38 for DECT. The uncertainties in the SPR extracted from SECT for soft tissue, and bone were found to be 1.4%, and 2.2%, respectively (Supplementary Table S1). The uncertainties in the SPR extracted from DECT for soft tissue, and bone were found to be 1.0%, and 3.8%, respectively (Supplementary Table S2).

The difference in SPR between SECT and DECT (*ΔSPR*) was calculated using the following equation.

6 $$\begin{array}{c}\Delta SPR={SPR}_{DECT}-{SPR}_{SECT}\end{array}$$

where, *SPR*_*SECT*_ and *SPR*_*DECT*_ are the SPR in SECT and DECT, respectively.

### Image acquisition

All measurements for DECT were performed with an Aquilion LB CT (Canon Medical Systems Corporation, Tokyo, Japan) scanner. CT images in a 512 × 512 matrix were reconstructed at a 2-mm slice thickness with a strong noise reduction level of the iterative dose reduction 3D (AIDR 3D) at the reconstruction kernel (FC13). The strong noise reduction level was used to be close to an image noise level to SECT. The effective tube current–time products of the low- and high-kV (80 and 135 kV, respectively) tubes were set to 543 and 509 mAs, respectively, to give a volume-weighted CT dose index (CTDI vol) of 28.3 and 35.8 mGy, respectively.

For ordinary image acquisitions (included as a comparator), SECT was performed using the same scanner operated at 120 kV with 591 effective mAs (CTDI vol = 47.4 mGy). SECT images were reconstructed using the mild noise reduction level of AIDR 3D at FC13 convolution kernel.

For DECT and SECT, the SFOV was set to 400 mm for head configuration of MODEL 062M and head and neck of the Rando phantom, and 550 mm for the other tested materials.

### Carbon-ion therapy treatment plans for the anthropomorphic phantom

The TPS used in this work was VQA Plan version 5.8 (Hitachi, Ltd., Tokyo, Japan)^15,23,39–42^. The TPS calculates the physical dose using an analytical dose calculation algorithm, a pencil-beam^43^ model with a triple Gaussian form^44^ for the lateral dose distribution, and a beam splitting algorithm^45^ to account for the lateral heterogeneity in the medium. The mixed beam model^46^ was implemented as the relative biological effectiveness (RBE) model in calculating the RBE of the scanned carbon-ion beam. These algorithms were used for optimization and clinical dose calculations.

**Figure 2:**
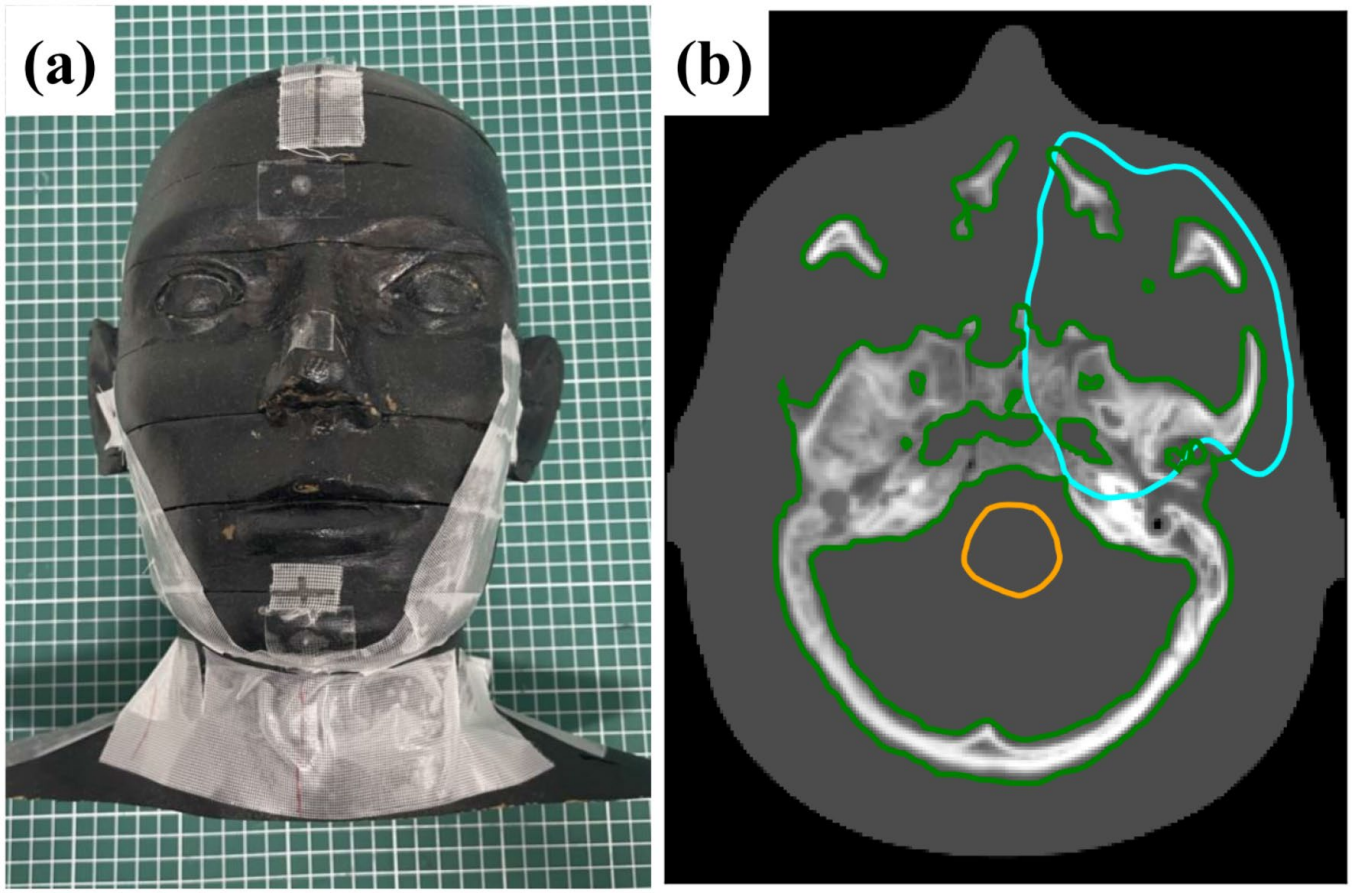
(**a**) A view of Rando phantom used for carbon-ion therapy treatment planning. (**b**) An example CT image with the clinical target volume (CTV) denoted as a light blue line located in the paranasal sinuses. The brain stem is drawn in orange as an organ at risk (OAR).

SECT and DECT scans of the cranial region of the Rando phantom were acquired to assess the differences in dose between SECT- and DECT-based carbon-ion treatment planning with human geometries. A carbon-ion therapy plan was created for a tumor in the anthropomorphic phantom to accommodate bony structures. Figure 2 shows a CT image of the clinical target volume (CTV) denoted as a light blue line located in the paranasal sinuses to imitate cancer. The treatment plan consisted of a left-angle field (the horizontal port with a couch angle of 0°) with a pencil beam of carbon-ion energy ranging from 73.7 to 227.3 MeV/u. The carbon-ion therapy plan was created with a single field uniformed dose (SFUD) such that a prescribed dose of D_95%_ (the dose to 95% of the CTV) = 64 Gy(RBE)/16 fractions was given to the CTV on SPR images with the SECT-SPR conversion. A forward calculation with the spot pattern (spot energy, spot position, and monitor unit) that was optimized for the SECT-SPR case was performed in the SPR image of DEEDZ-SPR conversion to investigate how the dose calculations affected the SPR conversion method. To only investigate the dosimetric impact of the SPR conversion methods in porous bone tissues, the SPR of the synthetic isocyanate rubber region (simulating soft tissue) was overwritten with an SPR of 1.0 for both the SECT- and DECT-based SPR conversion method.

Notably, the VQA plan used in this study executes carbon-ion dose calculations that are based on a specific look up table (LUT) from CT numbers to SPRs at 120 kV. Additional intermediate processing of the transformation from estimated SPR data to “hypothetical” planning CT data was performed to import the information of the estimated SPR into the TPS without altering the default LUT because only one LUT for one SFOV can be defined in the TPS. The estimated SPR images derived by the DEEDZ-SPR conversion were transformed into hypothetical CT images as if they were acquired at 120 kV by applying the “inverse function” of the specific LUT at 120 kV.

## Results

### SPR estimation

**Figure 3:**
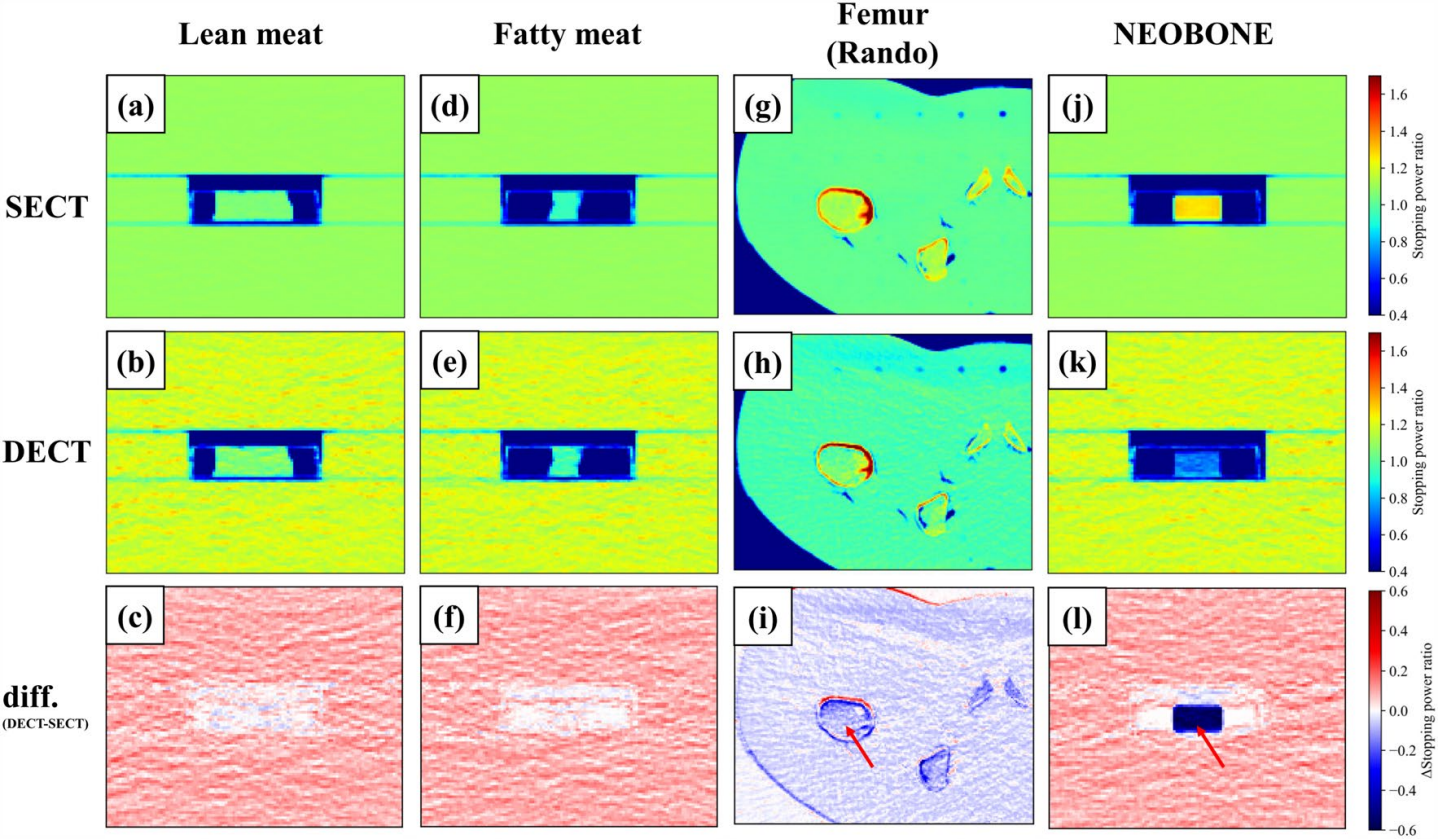
SPR images of examined samples: (**a**–**c**) lean meat, (**d**–**f**) fatty meat, (**g**–**i**) femur (Rando), and (**j–l**) NEOBONE. Upper, middle and lower panels indicate SECT-SPR, DEEDZ-SPR conversions and differential SPR distributions (DECT-SECT), respectively. Few differences were present in the SPR of lean meat and fatty meat between the SECT-SPR and DEEDZ-SPR conversions, whereas large differences were present for SPR of the femur of Rando phantom and NEOBONE between the SECT-SPR and DEEDZ-SPR conversions (red arrow).

**Figure 4:**
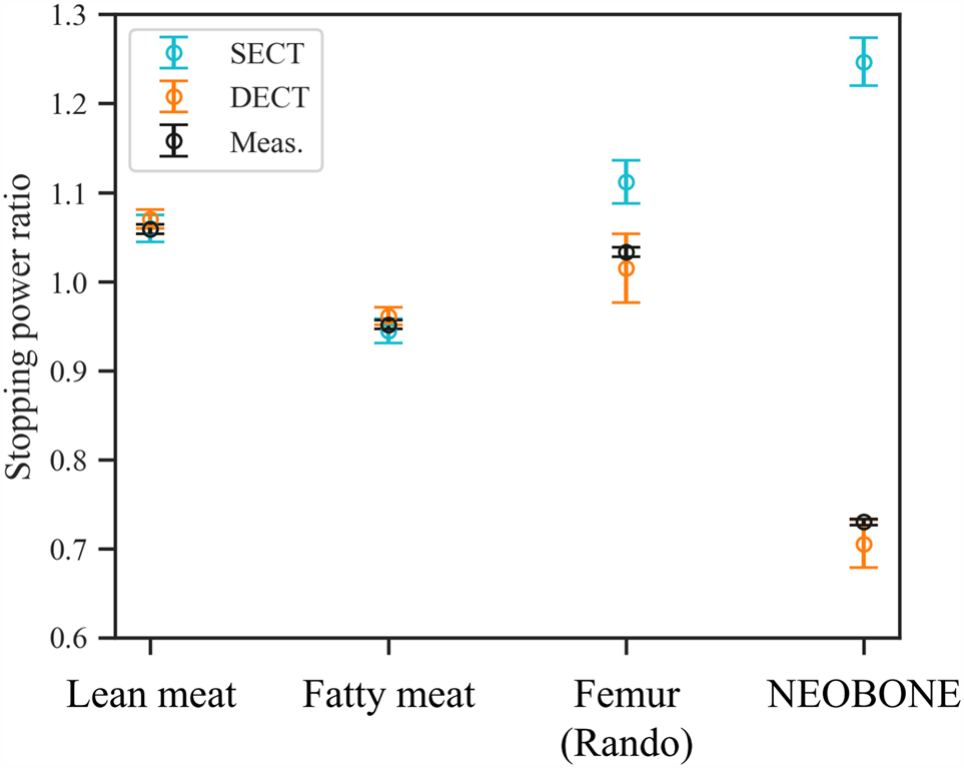
Comparison of SPR derived from SECT-SPR and DEEDZ-SPR conversions of each material to measured values. The bony materials showed large differences of SPR between the SECT-SPR and DEEDZ-SPR conversions. The error bars indicate the estimation uncertainties of the SECT-SPR and DEEDZ-SPR conversion methods and the measurement uncertainty of the SPR value.

The determined fit parameters and used parameters of the DEEDZ-SPR conversion using MODEL 062M are shown in Table 1. SPR images are shown in Fig. 3. Minimal differences were present in the SPR of lean meat and fatty meat (1.1% and 1.0%, respectively), whereas large differences were present in the SPR of the femur of the Rando phantom and NEOBONE between SECT-SPR and DEEDZ-SPR conversions. A comparison of SPR derived from SECT-SPR and DEEDZ-SPR conversions of each material to the measured values is shown in Fig. 4. The bony materials showed large differences in SPR between the SECT-SPR and DEEDZ-SPR conversions. The SPR estimated from DEEDZ-SPR conversion was closer to the measured value compared to the SECT-SPR estimation. For DEEDZ-SPR, SPR differences of − 1.8% and − 3.3% were seen for the femur of the Rando phantom and NEOBONE, respectively, whereas the differences for SECT-SPR were 7.6% and 70.7%, respectively.

**Table 1:** Determined fit and used parameters of the DEEDZ-SPR conversion. The c0 and c1 were taken from the paper^29^.

			R^2^
Low energy		80 kV	–
High energy		135 kV	–
α		1.01	
a		1.01	0.9998
b		1.00	
γ_L_		10.85	0.9557
c1	Soft tissue	0.3423	–
	Bone tissue	0.0696	–
c0	Soft tissue	0.0206	–
	Bone tissue	0.0444	–

### Carbon-ion dose calculations for treatment planning

**Figure 5:**
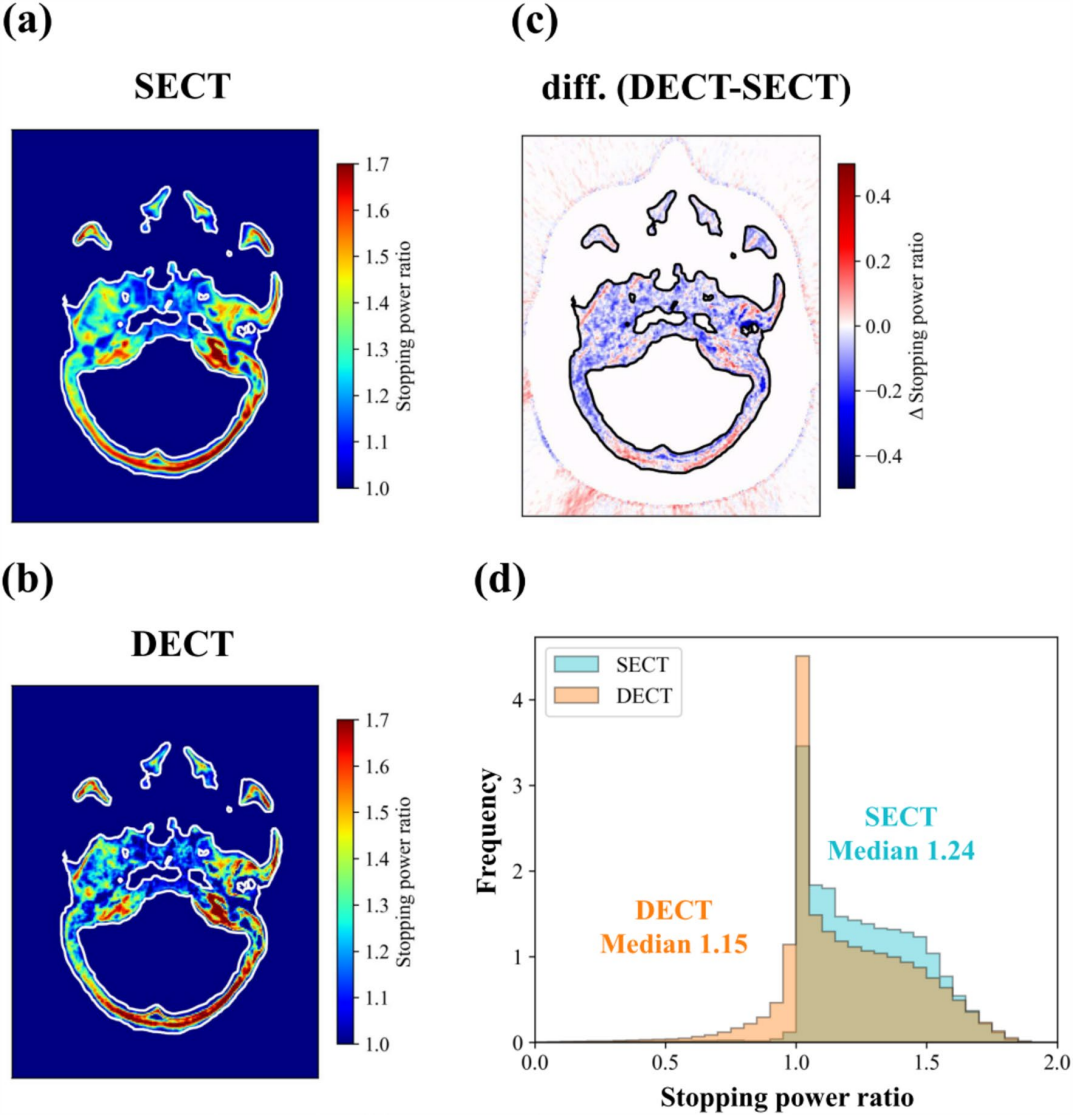
SPR images in bony regions between (**a**) SECT-SPR and (**b**) DEEDZ-SPR conversion images of the Rando phantom. (**c**) Subtraction image in bony regions between SECT-SPR and DEEDZ-SPR conversion images. (**d**) Histogram of the SPR within the bony structures from SECT-SPR conversion and DEEDZ-SPR conversion images.

The subtraction images of SPR in bony regions between the SECT-SPR and DEEDZ-SPR conversion images of the Rando phantom are shown in Fig. 5. The SPR derived from the DEEDZ-SPR was lower than that derived from the SECT-SPR conversion within the bony structures. The histogram of the SPR from DEEDZ-SPR was shifted towards lower values compared to the histogram for the SECT-SPR conversion, indicating the median of SPR estimated by DEEDZ-SPR conversion was 7.3% lower than that of the SECT-SPR conversion.

**Figure 6:**
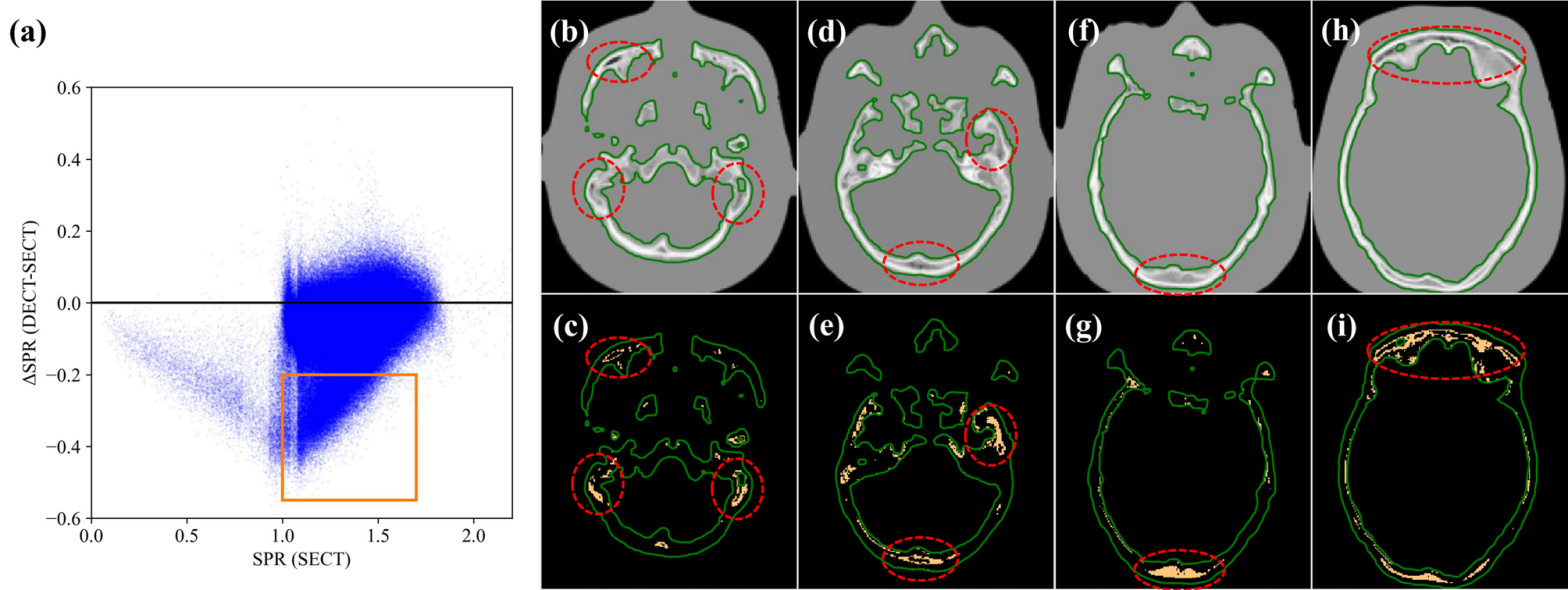
(**a**) Scatter plot of SPR difference between the SECT-SPR and DEEDZ-SPR conversions against the SPR by the SECT-SPR conversion. The region in the orange square in the scatter plot indicates a large difference in SPR that could cause a dose distribution difference between the SECT-SPR and DEEDZ-SPR conversions. Representative CT images in the axial plane (**b**, **d**, **f**, and **h**) and orange-hatched region with red-dotted ellipse on the representative CT images corresponds to the region in the orange square in the scatter plot where the bone structure contains a high density of pores (**c**, **e**, **g**, and **i**).

Figure 6 illustrates a scatter plot of the SPR difference between the SECT-SPR and DEEDZ-SPR conversions against the SPR by the SECT-SPR conversion. The region in the orange square in the scatter plot indicates a large difference in SPR. The orange-hatched region on the CT image corresponds to the region in orange in the scatter plot where the bone structure contains a high density of pores.

**Figure 7:**
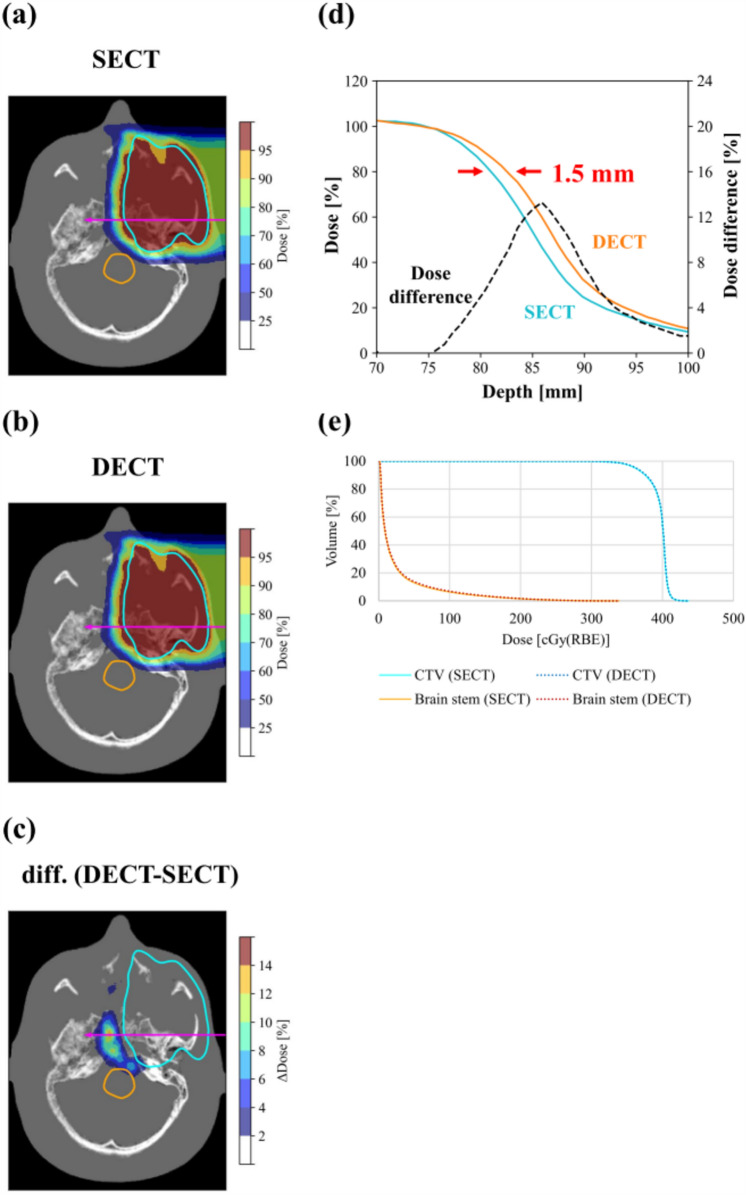
Transaxial dose distributions for the tumor located in the paranasal sinuses of SPR images acquired with (**a**) the SECT-SPR conversion and (**b**) the DEEDZ-SPR conversion. (**c**) Corresponding differential dose distributions (DECT-SECT), which were defined as the relative difference, are also presented. The CTV is marked as a light blue contour in each image. (**d**) A line-dose profile in the carbon-ion beam direction, indicated by the magenta line in each dose map, is provided. The estimated values of the carbon-ion range shift in tissue, which was defined as the difference of distal ranges at 80% of the prescribed dose between line-dose profiles obtained, is given in the graphs. (**e**) Dose-volume-histogram of the dose distribution between SECT-SPR conversion and DEEDZ-SPR conversion was shown.

Figure 7 demonstrates the effect of SPR differences between SECT-SPR and DEEDZ-SPR conversions on dose distribution. The range calculated with the DEEDZ-SPR conversion was longer than that calculated with the SECT-SPR conversion, illustrating a 1.5-mm shift of the range and a dose difference of 13.3% at maximum point in the evaluation of the dose distribution. The dose-volume-histogram (DVH), however, was almost the same between the SECT-SPR and DEEDZ-SPR conversions.

## Discussion

This study demonstrated the efficacy of SPR conversion methods derived from SECT and DECT for estimating SPR values in porous bone tissues with large density differences and the effect of the estimated SPR accuracy on the dose distribution of scanned carbon-ion beam therapy. The DEEDZ-SPR conversion method, which is based on DECT, were in better agreement with measurements than SECT-SPR for the bone tissues allowing an accurate range for the carbon-ion beams in carbon-ion dose calculations.

The SECT-SPR and DEEDZ-SPR conversions can accurately estimate the SPR of soft tissues in agreement with the measured SPR values (about 1%) as shown in Figs. 3 and 4. These results are similar to those of other studies (within ± 1%)^16–19^. The DECT-SPR conversion method could have an advantage in estimating SPR for porous bone tissues, especially when these include pores, regardless of the density of the pores. This was confirmed with SPR estimation for the femur of the Rando phantom and NEOBONE that showed that the SPR calculated with the DEEDZ-SPR conversion was close to the measured SPR values (Figs. 3 and 4). The SECT-SPR conversion could not consider the CT number of bone porosity because this did not reflect a change in density or chemical composition of an imaged material^7^ in addition to not reflecting the difference in patient size to that of the calibration phantom (i.e., beam-hardening effect)^10^. A trabecular bone contains bone marrow, and the heterogeneity of the density could be small for SECT- and DEEDZ-SPR conversion. However, porous bone with large density differences, such as the mastoid hive that is located upstream of the brainstem in the beam direction, would affect the SPR estimation in the SECT-SPR conversion but not in the DEEDZ-SPR conversion. The difference between human and animal bones may obscure the results caused by this^16^. This study used NEOBONE that is structurally close to human bone and the Rando phantom that uses human bone. Our approach would ensure accuracy of DECT-SPR estimation compared with other studies that used animal bones^16,17,19^. Even in human bone, differences in bone composition between adults and children have been observed^47^. A SECT-SPR conversion specified for adults would cause a SPR underestimation in the bone of approximately 5% for children younger than 6 years while a DECT-SPR conversion would not. Notably, DECT could improve the SPR estimation of bone with senile osteoporosis.

As shown in Fig. 5, the SPR derived from the DEEDZ-SPR was lower than that derived from the SECT-SPR conversion within the bony structures. In addition, the region in the orange square in the scatter plot indicates a large difference in SPR (Fig. 6a). These observations could produce a dose distribution difference between the SECT-SPR and DEEDZ-SPR conversions. In clinical practice, an uncertainty of 3.5% is considered for SECT-SPR estimation^15,36^. The observed 1.5-mm difference in the beam range with SECT-SPR conversion (Fig. 7d) is not considered in this uncertainty which means that the beam range difference would not be compensated with by this. The dose distribution derived from SECT-SPR conversion could overestimate SPR, especially in the structural bony regions, to cause beam stopping at points a few millimeters deeper than those estimated. This is critical for regions where organs at risk (OARs) are located downstream of porous bone as mentioned above. However, DEEDZ-SPR conversion can reduce the SPR uncertainty caused by the bone porosity that could contribute to more accurate range estimation and dose distribution calculation with scanned carbon-ion beam therapy. This advantage could contribute to the reduction in the range of uncertainty margins^48^. It is worthwhile to say that, with the estimated uncertainties in the SPR extracted from DECT (Supplementary Table S2), the range uncertainties could be reduced by about 1% in our DECT imaging.

This study has implications for the use of DECT-SPR estimation, and the observed results were not influenced by DECT acquisition techniques (e.g., Dual Source, Dual Spiral, TwinBeam, Dual Layer Detector, and Fast kV-switching)^49^. We only studied one DECT-SPR estimation method (i.e., DEEDZ-SPR conversion). Several other DECT-SPR estimation techniques exist, and these may give different results for SPR estimations. The SPR of the beef femur was overestimated by the SECT-SPR estimation, where a relative SPR error of 8.7% was obtained while the relative SPR error was only − 0.2% with the syngo.via Rho/Z algorithm^16^. Measurements with heterogeneous animal (pig) samples showed a clear reduction of the bias in range predictions in the presence of bones, with 0.88% for SECT versus 0.58% and 0.14% for the stoichiometric calibration method for DECT and Bayesian eigentissue decomposition method with DECT, respectively^17^. However, these studies did not include the porous bone examined in this study; generally, DECT-SPR estimation in bony regions can reduce the SPR estimation errors compared with SECT-SPR estimation. Further improvement of the SPR estimation can be expected with DECT-SRR estimation for largely heterogeneous bone porosity.

Further studies are needed to investigate the accuracy of SPR estimation against the different degrees of bone porosity and the effect of the estimated SPR accuracy on beam range estimation in patient data. For accurate dose calculation and especially for scanned carbon-ion beams, the current dose calculation does not include nuclear reactions. A method has been reported that corrects the dose against nuclear reactions with SPR^50^. The DECT-SPR conversion could improve the nuclear reaction correction caused by the accurate SPR estimation. A routine clinical use of DECT-SPR prediction requires full integration into the clinical workflow, including a function for DECT-SPR conversion and supporting the calculated SPRs in the TPS.

## Conclusions

In conclusion, this study demonstrated the efficacy of SPR conversion methods derived from SECT and DECT for estimating SPR values in porous bone tissues and the effect of the estimated SPR accuracy on the dose distribution of scanned carbon-ion beam therapy. The DECT-SPR conversion method was in better agreement with measurements than SECT-SPR for the porous bone tissues while SECT-SPR conversion overestimates the SPR in structural bony regions. DECT-SPR conversion is a promising method for accurate dose calculation in scanned carbon-ion beam therapy.

### Supplementary Information

Supplementary Information.

## Data Availability

The datasets used and/or analyzed during the current study are available from the corresponding author on reasonable request.
