# Supplementary material for: Dosimetric impact of stopping power for human bone porosity with dual-energy computed tomography in scanned carbon-ion therapy treatment planning
Source: Sci Rep. 2024 Jul 29;14:17440. doi: 10.1038/s41598-024-68312-y (PMC11286828; doi:10.1038/s41598-024-68312-y)
Supplement: Supplementary file 1 — Supplementary Information. [file 41598_2024_68312_MOESM1_ESM.docx]

**Supplementary Information**

Dosimetric impact of stopping power for human bone porosity with dual-energy computed tomography in scanned carbon-ion therapy treatment planning

Masashi Yagi et al., 2024

This file contains Supplementary Figure S1, and Tables S1-S2.

Supplementary Figure S1 CT number-to-SPR curves for head (blue) and body (orange) regions. The markers indicate the representative tissues used to calibrate the curves.

Supplementary Table S1. Uncertainties (1σ) in SPR estimation caused by different uncertainty sources for SECT.

|  | SPR estimation uncertainties (1σ) | | |
| --- | --- | --- | --- |
| Uncertainty source | Lung (%) | Soft (%) | Bone (%) |
| Uncertainties in patient CT imaging | 1.1 | 0.3 | 0.8 |
| Uncertainties in the parameterized stoichiometric formula to calculate theoretical CT numbers | 6.5 | 0.6 | 0.3 |
| Uncertainties due to deviation of actual human body tissue from ICRU standard tissue ^1^ | 0.2 | 1.2 | 1.6 |
| Uncertainties in mean excitation energies ^1^ | 0.2 | 0.2 | 0.7 |
| Uncertainty due to ignorance of SPR change with carbon energy by most commercial treatment planning systems | 0.2 | 0.4 | 1.0 |
| Total (root-sum-square) | 6.6 | 1.4 | 2.2 |

Supplementary Table 2. Uncertainties (1σ) in SPR estimation caused by different uncertainty sources for DECT.

|  | SPR estimation uncertainties (1σ) | | |
| --- | --- | --- | --- |
| Uncertainty source | Lung (%) | Soft (%) | Bone (%) |
| DECT imaging uncertainty | 2.2 | 0.8 | 3.6 |
| DECT modeling uncertainty | 1.6 | 0.3 | 0.3 |
| DECT inherent uncertainty | 0.1 | 0.4 | 0.2 |
| Uncertainty in the determination of mean excitation energies ^2^ | 0.2 | 0.2 | 0.6 |
| Uncertainty due to ignorance of SPR change with carbon energy by most commercial treatment planning systems | 0.2 | 0.4 | 1.0 |
| Total (root-sum-square) | 2.8 | 1.0 | 3.8 |

**References**

1 Yang, M. *et al.* Comprehensive analysis of proton range uncertainties related to patient stopping-power-ratio estimation using the stoichiometric calibration. *Physics in Medicine & Biology* **57**, 4095 (2012).

2 Li, B. *et al.* Comprehensive analysis of proton range uncertainties related to stopping-power-ratio estimation using dual-energy CT imaging. *Physics in Medicine & Biology* **62**, 7056 (2017).
